# A Review of the State of the Art in Non-Contact Sensing for COVID-19

**DOI:** 10.3390/s20195665

**Published:** 2020-10-03

**Authors:** William Taylor, Qammer H. Abbasi, Kia Dashtipour, Shuja Ansari, Syed Aziz Shah, Arslan Khalid, Muhammad Ali Imran

**Affiliations:** 1 James Watt School of Engineering, University of Glasgow, Glasgow G12 8QQ, UK; Qammer.Abbasi@glasgow.ac.uk (Q.H.A.); kia.dashtipour@glasgow.ac.uk (K.D.); shuja.ansari@glasgow.ac.uk (S.A.); muhammad.khalid@glasgow.ac.uk (A.K.); Muhammad.Imran@glasgow.ac.uk (M.A.I.); 2 Centre for Intelligent Healthcare, Coventry University, Coventry CV1 5RW, UK; syed.shah@coventry.ac.uk

**Keywords:** COVID-19, population health, Sars-Cov-2, AI, ML, disease diagnostics, sensing

## Abstract

COVID-19, caused by SARS-CoV-2, has resulted in a global pandemic recently. With no approved vaccination or treatment, governments around the world have issued guidance to their citizens to remain at home in efforts to control the spread of the disease. The goal of controlling the spread of the virus is to prevent strain on hospitals. In this paper, we focus on how non-invasive methods are being used to detect COVID-19 and assist healthcare workers in caring for COVID-19 patients. Early detection of COVID-19 can allow for early isolation to prevent further spread. This study outlines the advantages and disadvantages and a breakdown of the methods applied in the current state-of-the-art approaches. In addition, the paper highlights some future research directions, which need to be explored further to produce innovative technologies to control this pandemic.

## 1. Introduction

Since late 2019, countries around the world have been experiencing a global pandemic through the surfacing and spread of the potentially fatal COVID-19 (COronaVIrusDisease 2019) caused by SARS-CoV-2 (Severe Acute Respiratory Syndrome CoronaVirus 2) virus [1]. COVID-19 causes victims to develop a fever and display respiratory difficulties causing coughing or shortness of breath [2,3,4]. Data collected from victims of the virus shows that most deaths occurred in patients with underlying health issues with elderly people being at a higher risk of death [5]. The first confirmed case of the virus is considered to be in Wuhan, China in December 2019 with some of the early cases thought to be traced to seafood markets trading live animal species such as bats and snakes [6,7,8,9]. The virus has been discovered to likely be related to bats. It is suspected that the virus may have been transmitted to humans through bats which were being sold as food items [10,11]. The exact cause of the virus is still unknown, and it has also been suggested that the virus could originate from pangolins, which are natural hosts of corona viruses [12]. Pangolin is unlikely to be linked to the outbreak as the corona viruses found on the animal differ to COVID-19 [13]. However, it is possible the pangolin could have served as an intermediate host. As a result, these markets were shut down in China [14]. The virus rapidly spread throughout China and eventually spread throughout the world. The virus was officially declared a global pandemic by the World Health Organisation (WHO) on 30th January 2020 [15,16]. Although new discoveries are being made at the time of writing this paper, the virus has been found to be highly contagious and this has led to its rapid spread throughout the world [17]. The virus is spread primarily through respiratory droplets from an infected person [18]. These droplets can be dispensed by an infected person when coughing or sneezing. The droplets can then infect others directly via the eyes, mouth or nose when they are within a one or two meters radius of an infected person [19]. Some examples can show where 2 m is not enough distance such as with tobacco smoke traveling over 9 m from a lung source [20]. This uncertainly has led to the recommendation of using facial masks as a protective measure. There is debate on the effectiveness of masks, but it is recommended by the WHO to use masks if in contact with COVID-19 patients [21,22]. The droplets can also be passed to others indirectly due to their long-term presence on surfaces [23]. Another leading factor in the rapid spread is that those infected with COVID-19 can be contagious during the early stages of infection while they are showing no symptoms [24]. This leads to people believing they are not sick while unknowingly spreading the virus. One of the main challenges of the COVID-19 pandemic is the how the spread of the virus can be controlled. The rapid spread of COVID-19 has highlighted how the world’s population interacts when faced with a pandemic [25]. Governments around the world have outlined guidelines to their citizens to adhere to lockdown rules. Currently, the best strategy to control the spread of COVID-19 is to ensure social distancing until a vaccine or an effective treatment can be produced [26,27]. The National Health Service (NHS) of the United Kingdom is expecting an increased demand for their services as more COVID-19 patients are admitted and staff sick leave increases as staff members contract the disease [28]. Technology is being rapidly introduced in healthcare applications to develop systems that can ease the demand of the health service [29,30,31]. Any assistance via healthcare technology will free up valuable clinical resources to focus on other areas of care. In this paper, we look at the state-of-the-art non-contact sensing techniques and how these technologies can be used to assist in the care and detection of people suffering from COVID-19 and how these methods can help to reduce the spread of the disease, primarily the spreading of the disease from patients to healthcare workers such as doctors, nurses, and career staff.

### Search Strategy

The following search terms and variation of search terms were used in Google Scholar, MDPI, Science Direct and IEEE databases: radar breathing detection tachypnea, RGB-thermal breathing detection, Terahertz COVID-19, ultrasound non-contact lungs, ultrasound imaging, CT Scanning COVID-19, X-ray COVID-19, Camera COVID-19 Detection, Radar COVID-19 diagnosis, Thermography COVID-19, Terahertz COVID-19 detection, thermography non-contact, COVID-19 symptoms, Ultrasound Non-contact.

## 2. Non-Contact Sensing to Detect COVID-19 Symptoms

Non-contact sensing is the ability to detect information without direct contact with a subject. In terms of healthcare, non-contact can be used monitor the human body without devices physically touching the body. Non-contact techniques are considered highly valuable in dealing with a highly infectious disease such as COVID-19, as contact may contribute to the spread of disease. This is because healthcare workers will not need to make physical contact with patients to enable the monitoring of the patient. Using wearable devices can cause risks to healthcare workers as they will need to have physical contact with patients to attach the device. Despite precautions being undertaken such as wearing gloves and face masks, there will be lower risk if contact with patients can be successfully removed completely. Healthcare sensing technologies aim to collect information from a person which can be processed by Artificial Intelligence (AI) to provide decision support or directly analyzed by a clinician to diagnose a disease or monitor existing conditions. The use of AI can help to relieve pressure on hospital staff while they work hard to manage resources during the global pandemic. Non-contact remote sensing technology can sense such healthcare markers without introducing anything to the body (e.g., wearable devices). Wearable devices can be uncomfortable for some which will entice users to remove the device and results in misplacement or damage [32]. The non-contact techniques can assist in the detection of COVID-19 and the care of patients suffering from COVID-19. This will allow for quick diagnosis and allow for healthcare professionals to make clearer judgements on the treatment of the patient and allow for quarantine action to be undertaken. Vital-sign monitoring can provide great assistance in the fight against COVID-19 for several reasons. These reasons include detection of irregular breathing patterns, which is a major symptom of COVID-19, but it can also monitor the health conditions of patients suffering with COVID-19. Although COVID-19 affects the respiratory system [33,34], it has also been shown to take effect on the cardiovascular system [16]. These non-contact methods can also monitor heartbeats and therefore provide a monitoring system of the patient cardiovascular system. It can be concluded that non-contact sensing that monitors these vital signs can be used to aid in the detection and treatment of COVID-19. Examples of non-contact techniques described in this paper include computed tomography (CT) scans, X-rays, Camera Technology, Ultrasound Technology, Radar Technology, Radio Frequency (RF) signal sensing Thermography and Terahertz. Table 1 details the advantages and disadvantages of each technique. These methods can be used with AI to help give diagnosis. Currently testing for COVID-19 is done by doing a swab test. The results of these tests are currently returned the next day, but may be delayed by up to 72 h [35]. The paper will provide a review of the state-of-the-art literature that is using these non-contact methods to be able assist patients suffering with COVID-19. Table 2 provides a summary table of the current literature contained within this review paper.

### 2.1. CT Scanning

An example of a non-invasive technique to detect COVID-19 is using computed tomography (CT) scans [47]. This process involves taking several X-ray images of a person’s chest to create a 3D image of the lungs. The images can be reviewed by professionals to look for abnormalities in the lungs. The professionals are trained to review the images and they can tell from the captured image what is normal tissue of the lungs and which part of the lungs look to be infected. Infection can lead to inflammation of the tissue which will be present in the CT images. This method has been used to look for pneumonia which is an infection of the lungs which can affect the lungs similarly to how COVID-19 has an effect on the lungs of a patient. The activity of COVID-19 in the lungs is more prominent in the later stages of infection; however, ultimately, research has shown that CT scans showed a sensitivity of 86–98% [48]. This technique is non-contact as nothing is directly introduced into the body of the patient. However if a patient has been found to be infected with COVID-19 then the surface of the CT scanning machine is likely to contain droplets of the infection dispensed by the patient. This will therefore need to be cleaned effectively to prevent the spread of the virus to another patient who will be tested using the CT scanner apparatus. It can be noted that cleaning of surfaces can be considered safer for healthcare workers than physical contact with a patient. This is because droplets that are present on surfaces are likely to be static, whereas infected patients will dispense these droplets from their bodies during breathing and possibly through coughing, which is a symptom of COVID-19. CT scans can achieve high precision with high image resolution, however the technology used to perform CT scans is expensive. CT Scanners are paid for out of hospital budgets and are part of the dedicated equipment used to assist hospital staff in patient diagnosis. Their cost is proportionate to the level of accuracy they can provide within the healthcare industry. The equipment is not portable, and it requires skilled professionals for image analysis. The CT scanning machine is a massive piece of equipment. The machine is big enough to scan the entire length of an adult laying down. This also ensures the machine is of a high weight which will further remove the portability of the device. Another disadvantage of CT scanning is that the patient is exposed to radiation [49]. The radiation levels in CT scans have been found to result in an estimated cancer mortality risk of 0.08% within a 45-year-old adult [50]. Recently, AI has been used on CT images for diagnosis of COVID-19 [51]. Again, AI can allow for support for the skilled professionals analyzing the CT images produced by the CT scanners. If AI can assist with the detection and predictions of any disease in the lungs, this can help to ease the workload of the CT scan professionals. The advantages of this can allow for greater care of patients and more opportunity to ensure the appropriate safety prosecutions are being taken to prevent the spread of COVID-19 to the hospital staff or other patients who could potentially be classed as at high risk of COVID-19.

**Figure 1:**
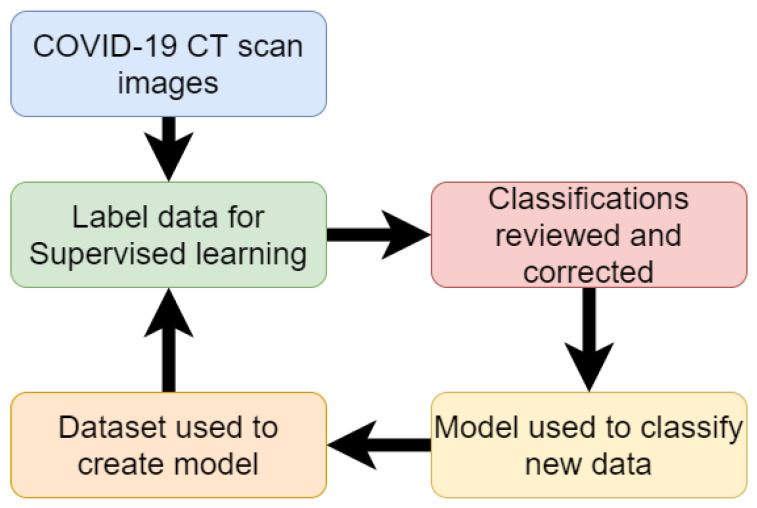
Flow chart of work for detection of COVID-19 from CT scan (Reproduced from [45]).

Fei Shan et al. [45] developed a deep-learning model which was able to detect COVID-19 and the level of infection within the lungs. Their model adopted a human-in-the-loop (HITL) strategy. Human-in-the-loop is when specialists are used to label a small amount of training data. Then an initial model is trained. Then this initial model is used to classify new data. The specialist then corrects any incorrect labels and the data set can be used to train further models. This task can be iterated numerous times to reduce the tedious task of labeling large amounts of data. The experiment used 249 confirmed cases of COVID-19 for training. The experiment achieved a high result of 91.6% accuracy. The experiments of this paper used 3 iterations. The first iteration made classifications on the validation data using 36 labeled images as a data set with an accuracy score of 85.1%. The labels are then corrected and added to the second iteration. The second iteration used 114 images for training and achieved an accuracy result of 91.0%. The labels are then corrected and passed to the third iteration. The third iteration is used on all 249 training images and achieved an accuracy result of 91.6%. The improved accuracy greatly reduces the human involvement and time devoted to labeling the full data. Figure 1 displays a flow chart of the process of human-in-the-loop.

**Figure 2:**
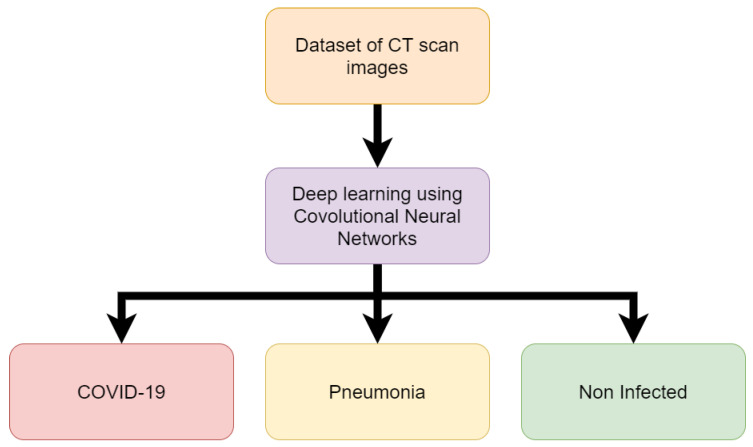
Flow chart of work for detection of COVID-19 from CT scan (Reproduced from [37]).

Li, Lin, et al. [37] used a COVNet, a custom deep-learning neural network to predict COVID-19 in CT images. The complete data set used included 400 COVID-19 CT images, 1396 Pneumonia CT images and 1173 non-infected CT images. The model takes CT images as input and extracts features of COVID-19 and pneumonia evidence found in the CT images. The features are then combined and the neural network can be applied to make predictions on whether the CT images contain COVID-19 or pneumonia features or if the CT images are of that of a non-infected person. Results found that the model was able to predict COVID-19 in patients with 90% sensitivity. The model proved to not only be able to detect infected and non-infected lungs but was also able to differentiate between COVID-19 and pneumonia with pneumonia having a sensitivity of 87%. Once the model was trained it was able to classify new samples within 4.51 s [37]. Figure 2 shows the process followed in this research.

**Figure 3:**
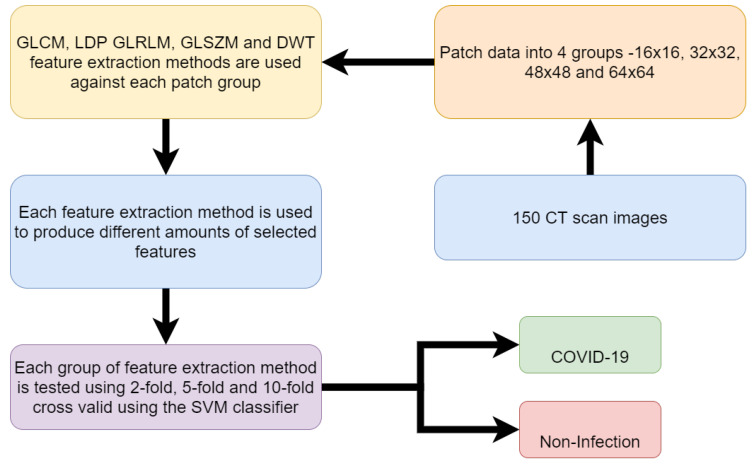
Flow chart of work for detection of COVID-19 from CT scan (Reproduced from [37]).

The research of Barstugan, Mucahid et al. [42] used machine learning on a data set of 150 CT images. The data set contains 53 infected CT images. Patches of the images are taken. Patches in image processing is the process of taking images and dividing them into containers of different sizes of pixels. Different sized patches are used to create 4 different samples of patches. The patch sizes are 16 × 16, 32 × 32, 48 × 48 and 64 × 64. The images were labeled as infected CT images and non-infected CT images in regard to COVID-19. The research used different methods of feature extraction on the images. These methods include Grey-Level Co-occurrence Matrix (GLCM), Local Directional Patterns (LDP), Grey-Level Run Length Matrix (GLRLM), Grey-Level Size Zone Matrix (GLSZM) and Discrete Wavelet Transform (DWT). Support Vector Machine (SVM) algorithm was then used to classify the extracted features of each of the methods. Support Vector Machine was used on the features using 2-fold, 5-fold and 10-fold cross-validation. Cross-fold validation is the process of using each fold to work as both training and testing data for the model to make predictions. Each fold will take a turn as being the testing data while the others are used as training. This is repeated for however many folds there are so that each fold serves as the testing data at least once. Then the results are compiled and each sample will have predictions made on it as it served as the testing data through each fold. The best accuracy result achieved out of the various methods of experimentation was 99.64%. This result was achieved using Discrete Wavelet Transform feature extraction method with 10-fold cross-validation using the 48 × 48 patch dimension CT images. A flow chart of the methodology followed in this research is shown in Figure 3.

The above papers have shown through experimentation that CT scanning can display the signs of COVID-19 within a person’s lungs. The research has also shown how AI can be used to make predictions of CT images and provide assistance in the determination of whether COVID-19 is present in the lungs or not. The studies have also shown that AI can determine the level of infection present in COVID-19 patients. The AI has also been able to differentiate between pneumonia and COVID-19 infections which is a positive as COVID-19 and pneumonia is similar in the way that both diseases attack the lungs. Table 3 provides a breakdown of the above research papers on using CT scans for non-contact COVID-19 diagnosis and care of patients.

### 2.2. X-Ray Imaging

X-ray images can provide an analysis of the health of the lungs and are used frequently to diagnose pneumonia [52]. The same strategy is used with X-ray images of the lungs to display the visual indicators of COVID-19 [53,54]. This is due to the similarities between COVID-19 and pneumonia as diseases that take an effect on the respiratory system. Similar to CT scans, X-ray equipment is also expensive and requires professionals to analyze the X-ray image.

The paper entitled “Automatic detection of coronavirus disease (COVID-19) using x-ray images and deep convolutional neural networks” used X-ray images taken of COVID-19-infected lungs and patients with lungs that were non-infected with COVID-19 to create a data set of x-ray images which was then used to predict COVID-19 automatically in patients. The X-ray images are passed into a ResNet-50 Convolutional Neural Network (CNN) which successfully obtained results of 98% accuracy in the differentiating between COVID-19 infected X-ray images and the non-infected x-ray images [38].

The paper of Zhang, Jianpeng, et al. [44] used deep-learning techniques on a data set of X-ray images of 70 patients confirmed to have COVID-19. Additional images of patients with pneumonia are added from a public chest X-ray image data set. The model is used to identify differences in X-ray images between patients infected with COVID-19 and patients suffering from pneumonia. The proposed deep-learning model was able to achieve a sensitivity of 90% detecting COVID-19 and a specificity of 87.84% in detecting non-COVID-19 cases.

**Figure 4:**
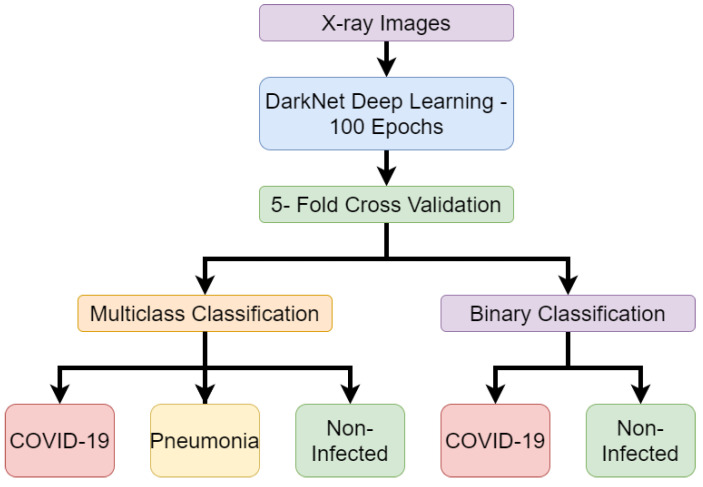
Flow chart of work for detection of COVID-19 from X-ray images (Reproduced from [39]).

Ozturk, Tulin, et al. [39] also conducted experiments using deep learning to classify X-ray images of patients with real-time classification of COVID-19. The experiments made use of a custom deep-learning model named DarkNet to perform binary and multi-class classifications. The binary classification is the process of deep learning, making predictions based on two choices. In the case of this experiment, the binary classification seeks to distinguish between COVID-19 and no findings of disease. Multi-class classification is when AI is tasked with making classifications on more than two possible classifications. This differs from binary classifications as the model must make decisions on which class data belongs to rather than just making distinctions between data. The multi-class classification distinguishes between no findings of disease and or if disease is found, and then whether the disease is pneumonia or COVID-19. The experiments used a publicly available data set of COVID-19 X-ray images and another publicly available data set for non-infected and pneumonia X-ray images. The complete data set included 127 COVID-19 X-ray images and 500 pneumonia X-ray images and 500 non-infected X-ray images. The deep-learning process made use of the developed DarkNet neural network. The complete X-ray image data set was divided between 80% training data and 20% testing data. The deep learning was run for 100 epochs using 5-fold cross-validation. Each epoch is an iteration of when the data is passed through the neural network. The neural network will learn about the data being passed through. Repeating epochs can allow for the model to fine-tune its biases and weights on what it believes data should be classified as. Then the model can improve the accuracy as it learns what works and does not until it can provide the best results obtained. The results produced an accuracy score of 98% for binary classification and an accuracy score of 87.02% for multi-class classification. It is expected that the result will fall as the number of classifications increase as the AI will need to recognize more features to distinguish between classes rather than differentiate between two data patterns. The complete process followed in this work is detailed in Figure 4.

### 2.3. Camera Technology

Camera technology can be used to provide non-contact sensing by observing the chest movements of an individual [55]. This can be achieved by capturing video footage of movements of the chest or, in the case of depth cameras, they are able to calculate depth by using two sensors with a known range [56]. The information captured using camera technology can be used provide assistance in the detection of COVID-19 as one of the symptoms of the disease includes an increase in the breathing rate of patients.

The paper “Combining Visible Light and Infrared Imaging for Efficient Detection of Respiratory Infections such as COVID-19 on Portable Device” used RGB-thermal camera footage for the detection of COVID-19. The footage was used with machine-learning binary classification to detect normal and abnormal breathing from people wearing protective masks. This research is relevant as masks are now commonly worn by people around the world as a preventive measure against COVID-19. The research collected real-world data and applied deep learning to achieve a high result of 83.7% accuracy which is the highest result found in the literature in regards to breathing detection using RGB-thermal imaging with deep-learning models. This research can provide a scanning method which can be used to control the spread of the virus and work with protective masks, thus reducing spread of COVID-19 [41].

**Figure 5:**
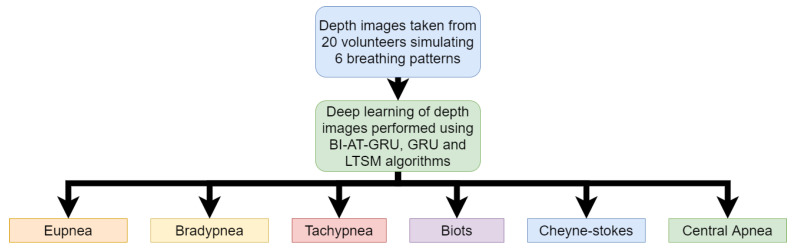
Flow chart of work for detection of COVID-19 from Depth Camera Image (Reproduced from [36]).

Wang, Yunlu, et al. [36] used Microsoft Kinect cameras to take depth images of volunteers breathing. A total of 20 volunteers were asked to sit on a chair and simulate 6 different breathing patterns. The breathing patterns were eupnea, bradypnea, tachypnea, biots, Cheyne –Stokes and central apnea. Each of these patterns display a different breathing rate in the individuals. Patients of COVID-19 display the rapid breathing pattern of tachypnea. During data collection, a spirometer was used to ensure the breathing pattern was being simulated correctly by the volunteers. The depth images taken using the camera were used in a deep-learning neural network model to classify the abnormal breathing patterns of tachypnea associated with COVID-19. The deep-learning model used was the BI-AT-GRU algorithm. Gated Recurrent Unit (GRU) is a simplified version of the Long-Term Short Memory (LTSM) algorithm. The BI-AT-GRU algorithm results achieved a high accuracy score of 94.5%. This research shows how depth images can be used to identify the tachypnea breathing patterns observed in COVID-19 patients in real time. The process map for this research is shown in Figure 5.

The primary disadvantage of using this method is the cost of thermal and depth cameras and the camera operators. Although the price of these cameras is falling gradually, it remains substantially high [57]. The cost of the equipment is of course less expensive than methods such as CT and X-ray scanning, but still more expensive than other methods discussed further in this paper. The research done with cameras has shown that the devices can be used with AI in the detection of COVID-19 and without contact with the body. This allows for more techniques to be implemented where diagnosis of COVID-19 can be achieved in a safe manner without increasing the risk of spreading the disease.

### 2.4. Ultrasound Technology

Ultrasound technology can be applied to detect respiratory failure of the lungs. An ultrasound machine is a device that uses high-frequency sound waves to image body movements [58]. The sound waves bounce off different parts of the body which create echoes that are detected by the probe and used to create a moving image. Lung ultrasounds have seen great development in recent years [59]. The use of ultrasound technology can be used in the detection of COVID-19 in a non-contact method where the risk of healthcare professionals becoming infected from patients can be decreased [60,61]. Ultrasound technology becomes contactless by using an ultrasound transmitter and receiver. Respiratory movement can then take place between the transmitter and receiver and creates a Doppler affect. This can then be used to create a contactless breathing monitor [62,63,64]. Ultrasound technology can be performed using smartphones for the signal and processing of ultrasound images in a portable setting [65]. The disadvantage of ultrasound technology is that patients must prepare themselves before an ultrasound can effectively create an image of the body [66]. This preparation can include not eating for a few hours before.

**Figure 6:**
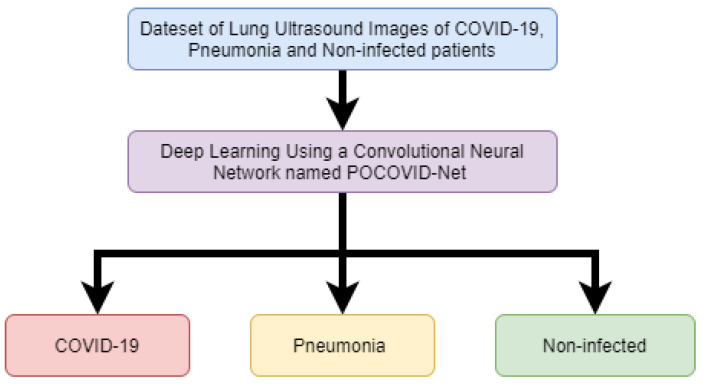
Flow chart of work for detection of COVID-19 from Ultrasound Technology (Reproduced from [46]).

The work of Born, Jannis, et al. [46] shows that ultrasound technology can be used in deep-learning models to distinguish the differences in COVID-19, pneumonia, and no infection within the lungs. The research collects a data set of lung ultrasound images which contain video recordings of lung ultrasound scans. The data set includes a total of 64 video recordings with 39 of the recordings of COVID-19 patients, 14 videos of pneumonia patients and 11 videos of non-infected patients. The paper has developed a deep-learning convolutional neural network named POCOVID-Net. The deep-learning algorithm was able to achieve an accuracy score of 89%. These ultrasound devices can diagnose 4 to 5 patients per hour. Figure 6 shows a simplified flow graph of the experiment undertaken in this paper.

### 2.5. Radar Technology

Radar technology can be used to monitor the respiratory system within a home environment and provide a quick response if abnormalities are found, which suggests COVID-19 being present. Radar systems use frequency-modulated continuous wave (FMCW) to observe the Doppler effect when a person moves [67,68,69,70]. This can be used to monitor the fine movements associated with breathing. This is achieved by using the images captured by the radar systems then applying AI to classify the images. AI models can be used to give real-time classification on new images [71,72,73]. Research done shows that radar technology can achieve 94% accuracy for the detection of breathing rates and 80% accuracy for heart-rate detection [34,74,75]. The Israeli military force has made use of radar systems for monitoring the vital signs of COVID-19 patients. The goal of using this method is to prevent medical staff from becoming infected while caring for patients [40,76]. Tachypnea is a symptom of COVID-19 and can be detected in a patient by using radar sensing technology [63,68,77]. Using radar technology to monitor vital signs can provide non-interference monitoring; however the disadvantage of radar systems is that it has high power requirements and the technology comes at a high cost [78].

### 2.6. Radio Frequency Signals

The use of radio frequency (RF) signal sensing can detect the vital signs of individuals by sensing the minute movements of the chest made while breathing as the heart beats ( [73,79,80,81,82]). This technique can be used for monitoring the vital signs of patients independent of their activities [83]. The RF signals detect the movement by observing the Channel State Information (CSI), which can show amplitudes of the RF signals while movement occurs between a RF transmitter and receiver [84,85]. The Emerald system has been developed to monitor COVID-19 patients using RF signals. The system uses RF signals to detect the breathing rate of COVID-19 patients and then uses AI to infer the breathing rate of the patient. This allows for doctors treating the patients to be able to monitor the patient from a safe distance. This method prevents the risk of infection to staff and provides the patient comfort as they do not need to wear monitoring devices [43]. RF signals have been used in previous research to detect breathing rates. RF signals can be used to detect abnormal breathing patterns such as tachypnea [86], which is a symptom of COVID-19 [36]. Systems have been developed to allow for real-time monitoring of breathing patterns using RF signals [87]. RF signals can be vulnerable to other movements within the room. The other movements create noise in the Channel State Information which can then in turn cause false readings [88,89].

### 2.7. Thermography

Thermography is a widely used non-contact technique within the medical community [90,91]. It has been used for mass screening of people in other pandemics such as H1N1 and Ebola so it can be applied in this current pandemic of COVID-19 [92]. Thermography works by using infrared radiation to calculate the temperature of the human body [93]. Abnormal body temperatures are a well-known indication of infection [94]. Symptoms of COVID-19 have been found to include high temperatures over the normal body temperature of 36–37 degrees Celsius [95,96]. Thermography can also be used to monitor the respiratory systems of patients and provide detection of breathing patterns such as bradypnea or tachypnea using AI [97]. Thermography has been recommended as an early detection strategy for COVID-19 among large amounts of people in places such as in airports [98]. Deep learning has been applied to thermal images where classifications on new images can be made in under a second [99,100].

### 2.8. Terahertz

Terahertz sensing technology is the process of directing terahertz beams to a person’s body to detect the motion of the chest created by a heart beating or lungs inhaling or exhaling breath [101,102]. Terahertz sensing is a non-contact method which can achieve superior penetration depth [103]. This can be helpful when penetrating a patient’s clothes. These terahertz systems can be produced in a similar fashion to how the radar imaging takes place, except with using terahertz waves and observing the Doppler effect of the Terahertz wave while a patient performs the breathing issue [104]. Terahertz waves refer to electromagnetic frequencies around 0.1–10 Terahertz(THZ) [103,105]. The use of terahertz can detect disease such as COVID-19 [106]. This will work similarly to the radar system with AI being used to make classifications on the images showing the Doppler effect of terahertz waves. Deep learning can be applied to these images and give fast classifications of new models once an AI model has been fully trained. Terahertz radiation is considered the first choice in radiation exploitation due to the non-harmful properties to living cells [107]. A terahertz spectroscopy is an example of a powerful tool in medical research and diagnosis used for analysis of human breath samples and it offers a low cost [108].

### 2.9. Comparison to Contact Methods

The methods discussed in this paper have looked at non-contact techniques for diagnosing COVID-19. Due to the nature of the disease, it has been widely acknowledged that reducing contact between people is the best action to reduce the spread. Therefore non-contact technologies for diagnosis are the preferred method. Wearable devices can also be used for monitoring vital signs [109,110]. This monitoring of vital signs can therefore be used to detect any displays of COVID-19 symptoms. Popular devices such as AppleWatch, FitBit and Oura ring are highly available and provide monitoring of the heart rate [111]. The Oura ring has been found to show changes in body temperature associated with COVID-19 and has led to several studies being conducted into the use of Oura rings in early detection of COVID-19 [112,113]. These technologies are known as personal health trackers and in terms of COVID-19 detection, these devices will be better for self-diagnosis. If these devices can inform users that they are displaying COVID-19 symptoms then the user can take action. Non-contact methods will serve healthcare workers better as they can provide assistance to patients while still reducing contact with the patient and thus reducing risk of infection.

### 2.10. Future Directions

This section will detail some of the future directions which may be suitable for expanding on the research presented in this paper. The research has highlighted how the detection of COVID-19 is possible using various techniques. This section will now discuss how this research can be taken further to work within real-life scenarios.

- One of the biggest challenges with CT scanning to diagnose COVID-19 is the lack of portability. This means that although the method is non-contact, its use still requires individuals to travel to a location where the machine is available. As the CT images can provide high resolution, the AI can be used for the detection of COVID-19. Therefore, future directions of this method should look to creating highly accurate models that can eventually lead to the automation of COVID-19 detection. This can allow for faster diagnosis, which can allow for more patients to be tested and increase availability of staff operating and analyzing CT scans.
- X-rays, similarly to CT scans, are not portable. Like CT scans, professionals are required to operate these machines and analyze the X-ray images. The research presented in this paper has shown that AI can be used to make predictions if COVID-19 is present in the lungs. This can be useful similarly to CT scans where AI can be applied to make the predictions and speed up the process. The more data collected, the more advanced the model will become. Perhaps initially the predictions will need to be confirmed by humans but eventually the checks can become less frequent. Since the research above has displayed an ability of AI to distinguish between not just COVID-19 and non-infected but also pneumonia at high accuracy, then the AI has proved to be capable of accurate classifications.
- Thermal and depth cameras can detect the irregular breathing patterns that are associated with COVID-19 symptoms. The issue here is that even though the camera can detect the irregular breathing pattern, it is unable to categorically define COVID-19 as the cause for individuals displaying the irregular breathing patterns. In a real-life situation, the camera method may be better suited to monitoring vulnerable people who are considered high risk from COVID-19. Then once the monitoring system has identified the irregular breathing patterns, an alarm can be raised with a career or family member. Then, appropriate action can be taken for greater accuracy such as diagnosis with CT scanning or X-ray scanning.
- Ultrasound technology can take moving images of the lungs and detect COVID-19. This can also be made portable by using mobile devices. AI can be applied to recognize if COVID-19 or pneumonia is present in the lungs. This research can be further applied to develop applications on a mobile device that can capture an ultrasound of the lungs then compare it to an AI model to predict if COVID-19 is present. Although not all phones may not have the necessary hardware to achieve this, the non-contact method can allow for others to be able to use the devices for diagnosis at a safe distance.
- Radar technology can identify the breathing patterns of individuals. Much like camera technology, the identification of breathing patterns can raise cause of concern but it cannot isolate COVID-19 as the sole cause. Radar technology can again be used to monitor individuals but due to the high costs it is more likely to be used as a monitoring system within a hospital and not a home environment.
- Any future directions should consider the use of RF signals to detect the breathing patterns which give indication of COVID-19 symptoms. The RF systems can be implemented inexpensively using existing WiFi technology present within many homes. This allows for the monitoring of individuals without the costs incurred in implementing radar or camera technologies highlighted in this paper.
- Thermography has shown in previous research to be able to detect body temperatures of large amounts of people in previous pandemics. Therefore, it can be implemented in mass screening in the current COVID-19 pandemic. With the use of thermography being able to detect respiratory issues, it is clear that these systems can also be implemented for COVID-19 detection.
- Terahertz can provide deeper penetration and detect smaller movements such as the chest movements while breathing. This can therefore be used in early detection of COVID-19. The earlier the disease is detected, the sooner isolation can begin and ensure that further spread is reduced.

## 3. Conclusions

The works listed in this paper have shown that COVID-19 can be detected using contactless techniques. Techniques such as CT scans and X-ray imaging provide high accuracy and high image resolution, but the cost of the equipment is high and not portable. Thermal and depth camera technology has been used to detect breathing patterns, which is associated with COVID-19 symptoms. However, these cameras are expensive and need to be operated by a professional. Radar technology is also able to detect breathing patterns but carries disadvantages of high operating expenses and capital expenditures. RF signals provide low cost and high accuracy as compared with other non-invasive technologies. The technologies can work on AI which can allow for skilled professionals to be available to assist in other areas of healthcare during the pandemic. The non-contact methods also protect healthcare workers from contracting the disease. The future direction of non-contact detection should look at the use of RF systems as the cost is cheap and it is easier to implement within a home environment in comparison to other methods. This gives the advantage of allowing the users to remain within isolation.

## Figures and Tables

**Table 1:** Summary of Non-Invasive Techniques.

Method	Accuracy	Cost	Time for Measurement	Time for Results	Harm to Body	Skills of Operators	Possibility of AI
CT	High	High	Moderate	Fast	Low	High	Yes
X-Ray	High	High	Moderate	Fast	Low	High	Yes
Camera	High	Medium	Real Time	Real Time	None	Medium	Yes
Ultrasound	High	Medium/High	Moderate	Medium	Low	High	Yes
Radar	High	High	Real Time	Real Time	None	Medium	Yes
RF	High	Low	Real Time	Real Time	None	Low	Yes
IR Thermo	High	Medium	Fast	Fast	None	High	Yes
THz	High	Medium	Fast	Fast	None	High	Yes

**Table 2:** Summary of Current Literature.

Title of Paper	Citation	Year	Key Themes	Authority
Abnormal respiratory patterns classifier may contribute to large-scale screening of people infected with COVID-19 in an accurate and unobtrusive manner	[36]	2020	The paper details that COVID-19 patients display tachypnea (Rapid breathing). The paper looks at taking depth images to identify the breathing patterns of volunteers using deep learning	Peer reviewed paper. 24 citations on Google Scholar.
Artificial intelligence distinguishes COVID-19 from community acquired pneumonia on chest CT	[37]	2020	CT scan images are used in a COVNet neural network to distinguish between COVID-19, Pneumonia and Non-infected scan images.	Peer reviewed paper. 157 citations on Google Scholar.
Automatic detection of coronavirus disease (COVID-19) using x-ray images and deep convolutional neural networks	[38]	2020	X-ray scan images are used in a ResNet-50 Convolutional Neural Network (CNN) to distinguish between COVID-19 and non-infected scan images.	Peer reviewed paper. 102 citations on Google Scholar.
Automated detection of COVID-19 cases using deep neural networks with X-ray images	[39]	2020	X-ray images are processed using the DarkNet neural network to test binary classification between COVID and Non-infected and multi-class classification between COVID, Pneumonia and Non-infected.	Peer reviewed paper. 22 citations on Google Scholar.
Can Radar Remote Life Sensing Technology Help to Combat COVID-19?	[40]	2020	Radar systems have been used to monitor the vital signs of patients in a contact less manner to protect healthcare workers	Paper uploaded on researchgate.net.
Combining Visible Light and Infrared Imaging for Efficient Detection of Respiratory Infections such as COVID-19 on Portable Device	[41]	2020	RGB-Terminal camera footage used in a BiGRU neural network model between healthy and ill.	Peer reviewed paper.
Coronavirus (COVID-19) classification using CT images by machine-learning methods	[42]	2020	CT scan images are used to experiment with various methods of feature extraction and deep learning algorithms to achieve the best results	Peer reviewed paper. 157 citations on Google Scholar. 157 citations on Google Scholar.
CSAIL device lets doctors monitor COVID-19 patients from a distance	[43]	2020	Radio Frequencies have been used to monitor the vital signs of patients in a contactless manner to protect healthcare workers	Article found on MIT Computer Science & Artificial Intelligence Laboratory website.
Covid-19 screening on chest x-ray images using deep-learning-based anomaly detection	[44]	2020	X-ray images are used with deep learning to identify if samples are COVID-19 or Pneumonia	Peer reviewed paper. 32 citations on Google Scholar.
Lung infection quantification of COVID-19 in CT images with deep learning	[45]	2020	CT scan images are used in deep learning to identify COVID-19. Human-in-the-loop technique is used to focus on increasing accuracy	Peer reviewed paper. 52 citations on Google Scholar.
POCOVID-Net: automatic detection of COVID-19 from a new lung ultrasound imaging data set (POCUS)	[46]	2020	Lung Ultrasound videos of COVID-19, Pneumonia and non-infected patients used deep learning for classification.	Peer reviewed paper. 2 citations on Google Scholar.

**Table 3:** Summary of CT Scanning works.

Citation	Training Data	Algorithms	Results
[45]	249 CT images of COVID-19 showing different levels of infection.	Custom Convolutional neural network (CNN) called “VB-Net”	91.6% Accuracy
[37]	400 COVID-19 CT images, 1396 Pneumonia CT images and 1173 non-infected CT images	Custom Convolutional neural network (CNN) called “COVNet”	90% sensitivity of COVID-19 samples.
[42]	150 CT images including 53 COVID-19 cases.	Support Vector Machine	99.64% Accuracy
